# Exploring the Utility of Long Non-Coding RNAs for Assessing the Health Consequences of Vaping

**DOI:** 10.3390/ijms25158554

**Published:** 2024-08-05

**Authors:** Ahmad Besaratinia, Hannah Blumenfeld, Stella Tommasi

**Affiliations:** Department of Population & Public Health Sciences, USC Keck School of Medicine, University of Southern California, M/C 9603, Los Angeles, CA 90033, USA; hanblum@gmail.com (H.B.); tommasi@med.usc.edu (S.T.)

**Keywords:** electronic cigarette (e-cig), gene regulation, non-coding RNAs, tobacco, vaping

## Abstract

Electronic cigarette (e-cig) use, otherwise known as “vaping”, is widespread among adolescent never-smokers and adult smokers seeking a less-harmful alternative to combustible tobacco products. To date, however, the long-term health consequences of vaping are largely unknown. Many toxicants and carcinogens present in e-cig vapor and tobacco smoke exert their biological effects through epigenetic changes that can cause dysregulation of disease-related genes. Long non-coding RNAs (lncRNAs) have emerged as prime regulators of gene expression in health and disease states. A large body of research has shown that lncRNAs regulate genes involved in the pathogenesis of smoking-associated diseases; however, the utility of lncRNAs for assessing the disease-causing potential of vaping remains to be fully determined. A limited but growing number of studies has shown that lncRNAs mediate dysregulation of disease-related genes in cells and tissues of vapers as well as cells treated in vitro with e-cig aerosol extract. This review article provides an overview of the evolution of e-cig technology, trends in use, and controversies on the safety, efficacy, and health risks or potential benefits of vaping relative to smoking. While highlighting the importance of lncRNAs in cell biology and disease, it summarizes the current and ongoing research on the modulatory effects of lncRNAs on gene regulation and disease pathogenesis in e-cig users and in vitro experimental settings. The gaps in knowledge are identified, priorities for future research are highlighted, and the importance of empirical data for tobacco products regulation and public health is underscored.

## 1. Introduction

Electronic cigarettes (e-cigs) are electronic nicotine delivery systems (ENDS) that simulate tobacco smoking [1,2]. E-cigs are battery-powered devices that heat a solution (e-liquid) containing varying concentrations of humectants, nicotine, and flavors to produce a vapor, which users inhale through a mouthpiece [1,3]. E-cig use is also known as “vaping”, and e-cig users are commonly referred to as “vapers” [4,5]. E-cigs have been promoted originally as safe and subsequently as a less-harmful alternative to tobacco cigarettes [6,7]. This perception has promulgated by the fact that e-cigs “heat” e-liquid to render inhalable vapor, unlike traditional cigarettes that burn tobacco leaves to generate smoke [1,7]. Importantly, chemical analyses of e-cig vapor and e-liquid have shown the presence of many of the same toxicants and carcinogens as those found in cigarette smoke, although generally at substantially lower levels [1,4,8]. Currently, e-cig use is widespread among adolescent never-smokers and adult smokers seeking a reduced-harm substitute for combustible cigarettes [2,6,7,9]. To date, however, the long-term health effects of vaping are largely unknown [7,8,10]. Investigating the biological consequences of e-cig use can clarify the health risks or potential benefits of vaping in adult former-smokers as well as in youth never-smokers who have taken up this controvertible habit [5,8].

Many toxic and carcinogenic compounds present in e-cig vapor and cigarette smoke exert their biological effects through epigenetic changes that can cause dysregulation of disease-related genes [11,12,13,14,15,16,17,18,19,20,21,22]. The toxic and carcinogenic constituents of e-cig vapor and cigarette smoke, such as carbonyl compounds, volatile organic compounds (VOC), free radicals, and heavy metals, can induce a wide range of DNA lesions [1,10], many of which may interfere with the epigenetic machinery, e.g., by inhibiting the binding of epigenetic enzymes to their targets [15,23]. There may also exist a competitive demand for the metabolism of the chemicals present in e-cig vapor and cigarette smoke [15] and the cofactors/cosubstrates required for the epigenetic modifying enzymes [23]. Moreover, oxidative DNA damage induced by e-cig vapor and cigarette smoke can affect transcriptional regulatory elements and other epigenetic modifications [15,24].

Long non-coding RNAs (lncRNAs) have emerged as prime regulators of gene expression in health and disease states [25,26,27,28]. There is a mounting recognition of lncRNA-mediated dysregulation of genes in human diseases, including tobacco-related diseases [29,30,31,32,33,34,35,36]. Also, growing evidence shows the modulatory effects of lncRNAs on gene expression in response to specific cues or external stimuli such as tobacco product use [37,38]. While extensive research has shown that lncRNAs regulate genes involved in the pathogenesis of smoking-associated diseases [39,40,41,42], the utility of lncRNAs for assessing the disease-causing potential of vaping remains to be fully explored. Determining how lncRNAs regulate disease-related genes in e-cig users can lead to the identification of novel biomarkers of exposure and early effects for vaping. These biomarkers will have significant utility for assessing the health risks or potential benefits of vaping relative to smoking. This information is urgently needed by the regulatory agencies, including the United States Food and Drug Administration (FDA), which has the authority to regulate the manufacturing, marketing, and distribution of tobacco products to protect public health [6,9,43].

There is limited but growing research on lncRNA-mediated gene regulation in biospecimens from e-cig users and in cells treated in vitro with e-cig aerosol extract [13,44,45]. The present review provides an overview of the evolution of e-cigs as a highly consequential ENDS product, describes trends in e-cig use, and discusses the competing views on the public health impact of vaping. While highlighting the importance of lncRNAs in cell biology and disease, it summarizes the existing research on the modulatory effects of lncRNAs on disease-related molecular pathways and gene networks in e-cig users and in vitro experimental settings. The gaps in knowledge are identified, priorities for future research are highlighted, and the importance of empirical data for tobacco products regulation and public health is underscored.

## 2. Search Strategy and Selection Criteria

PubMed search was conducted to identify references using the following terms: “vaping”, “smoking”, “tobacco”, “cigarette”, “electronic cigarette”, “e-cigs”, “electronic nicotine delivery systems”, “ENDS”, “youth vaping”, “long non-coding RNA”, “lncRNA”, and “non-coding RNA”. The search terms were used both individually and in combination with each other. All English-written references, published on or before 30 June 2024, were considered. Where appropriate, publicly available databases and scientific reports from regulatory agencies and/or academia as well as news publications were considered; in all cases, cited sources were identified with a link to the published materials. To limit the number of citations, updated reviews were used rather than individual research articles, unless otherwise indicated.

## 3. E-Cig Technology

E-cigs have been promoted as a safe or less harmful alternative to tobacco cigarettes and as an aid to smoking cessation [2,5,8]. E-cigs are handheld, battery-powered devices that heat a liquid and convert it into a vapor, which users inhale into their lungs [1,3,46]. The liquid, also known as “e-liquid/e-juice”, is a mixture of propylene glycol (PG), glycerin/vegetable glycerin (VG), flavorings, and varying concentrations of nicotine, although nicotine-free e-liquid is also available [1,10,46]. Since the introduction of e-cigs to the U.S. market in 2007, these devices have evolved rapidly and substantially, from the first-generation “cig-a-like”, which was designed to look like a traditional tobacco cigarette, to the second-generation vape pens, third-generation box mods, and the currently popular fourth-generation pod-based devices [1,10]. The fast and significant changes in the design and features of e-cig devices have been accompanied by massive diversification of e-liquids [47,48,49]. With hundreds of chemicals being added to e-liquids to produce appealing flavors to every man, woman, and child, there are numerous combinations of chemicals in the ~20,000 e-liquids currently available in the market [49,50,51]. The combination of these chemicals, when vaporized altogether, can give rise to many more chemicals with uncertain toxicity profiles [52,53,54,55,56,57,58,59].

## 4. Chemical Composition of E-Cig Liquid and Vapor

Chemical analyses of e-cig liquid and vapor have revealed the presence of some of the same toxicants and carcinogens as those found in cigarette smoke, although mostly at substantially lower levels—these chemicals include carbonyl compounds, VOC, free radicals, and heavy metals, among others [1,3,10,60,61]. E-cig vapor also contains chemicals that are not present in cigarette smoke [51,62]. The latter compounds likely arise from mixing of the e-liquid ingredients and/or vaporization of humectants (PG/VG), flavorings, or chemicals leached from the device components [53,63]. At least seven categories of harmful and potentially harmful substances have been identified in e-cig liquid and vapor, including carbonyls, VOC, nicotine, nanoparticles, trace metal elements, bacterial endotoxins, and β-glucans [10]. The reduced levels of toxicants and carcinogens in e-cig vapor are consistent with the fact that e-cigs, unlike conventional cigarettes, do not “burn” tobacco to produce aerosol for inhalation [3,4]. This has led, in part, to the perception that e-cig use is safe or less-harmful than tobacco smoking [2]. While the lower levels of toxicants and carcinogens in e-cig vapor may imply reduced health risks, they cannot, however, equate to no risk [64]. In fact, exposure to many of the same constituents of e-cig vapor, at varying concentrations, has been associated with a variety of diseases, including cardiovascular-, immune-related (inflammatory), and respiratory diseases and cancer, among others [1,3,10,15,23,65].

## 5. Safety, Efficacy, and Health Risks or Benefits of E-Cig Use

There are competing views on e-cigs safety, efficacy, and health risks/benefits [6,66,67,68,69]. On the one hand, advocates for e-cigs claim that vaping, especially when combined with counseling and behavioral therapy, helps smokers quit; meta-analyses of dozens of randomized clinical trials mostly support this claim [7,9]. An important caveat is that e-cigs as a medical intervention but not a consumer product may help adult smokers quit [2,9,70,71,72,73]. Opponents of e-cigs argue that numerous population-based studies [74,75,76] confirm that e-cigs as a consumer product are not effective for smoking cessation [7,9,77]. As it stands, e-cigs are not approved as a medical intervention anywhere in the world; instead, e-cigs are consumer products that can be bought if one is of a certain age—e-cigs can be used however one wishes, as much as one likes, and as frequently as one wants [76,78]. Proponents of e-cigs also claim that the use of e-cigs has led to a decline in youth smoking [2,79]. Opponents, however, counter that e-cigs are addicting a new generation of teens and youth who would have never experimented with smoking in the first place [68,78]. It is also argued that vaping may lead to smoking and “renormalization” of this unhealthy habit [72,80,81]. While advocates of e-cigs claim that e-cigs cause less harm than tobacco cigarettes, opponents disagree [6,66,68,78,82].

## 6. Disentangling the Biological Effects of E-Cig Use in Adult Vapers

Adult e-cig users are likely to have a prior history of smoking (i.e., ex-smokers/current vapers) or co-use e-cigs and combustible cigarettes (i.e., dual users) [2,6,9,64]. The existing literature on the “potential” health risks of vaping is often criticized by the fact that the study participants in many reports include adult e-cig users with “past” or “current” smoking habits (ex-smokers/current vapers or dual users, respectively) [4,67]. This has complicated the interpretation of the results, as it is less than certain whether the observed effects in e-cig users are due to (i) persistent effects of past smoking (in former smokers) or current smoking (in dual users), (ii) current vaping only, or (iii) a combination of the two [2,4,6].

## 7. LncRNAs: Mechanisms and Functions

Although the vast majority of the human genome is transcribed into RNA, only ~2% of this RNA is protein-coding [83,84,85,86]. The most abundant and diverse class of non-coding RNAs is lncRNAs [85,87]. LncRNAs (≥200 nucleotides in length) are increasingly recognized as versatile regulators of gene expression at the transcriptional, post-transcriptional, translational, or post-translational levels [28,88,89,90]. LncRNA are often classified into different sub-types, including intergenic (long intergenic noncoding RNAs (lincRNAs)), intronic, and sense and antisense lncRNAs, with each sub-type having different genomic position in relation to genes and exons [91,92]. The majority of lncRNAs are overlapping sense and antisense transcripts, which mostly include protein-coding genes [83], thus resulting in a complex hierarchy of overlapping isoforms [93]. Genomic sequences within these transcriptional foci are frequently shared within a number of coding and non-coding transcripts in the sense and antisense directions relative to annotated genes [94]. LncRNAs are also derived from “pseudogenes” that are abundantly present in the human genome. Some of the ~15,000 pseudogenes identified in the human genome [95] have been shown to be functional [96,97]. In mammals, lncRNAs are dynamically expressed at various stages of development [98] and during differentiation of stem, muscle, mammary gland, immune, and neural cells, among others [99,100]. There is a transition in lncRNA expression during development, with broadly expressed and conserved lncRNAs evolving into an increasing number of lineage-specific and organ-specific lncRNAs [101,102]. The regulatory function of lncRNAs is primarily attributed to their roles as (1) “signals” in response to different stimuli or combinatorial actions of transcription factors (TFs); (2) “guides” to recruit histone-modifying enzymes or chromatin modifiers to the positions of target genes either in *cis* or in *trans* action; (3) “decoys” or “sponges” to titrate TFs or other intermediary regulatory entities such as RNA/DNA molecules (e.g., microRNAs (miRNA)) and sequester them away from their respective target site; and (4) “scaffolds” to recruit protein partners together by forming functional ribonucleoprotein complexes [103,104,105,106,107,108]. Various sub-types of lncRNAs exert their regulatory effects through participation in competing endogenous RNAs (ceRNAs) regulation, transcription regulation, and epigenetic regulation [106,107,108]. There is a growing appreciation of lncRNA-mediated dysregulation of genes in human diseases, such as cardiovascular, respiratory, and immune diseases and cancer, among others [29,30,31,32,33,34,35,36]. Emerging data show the modulatory effects of lncRNAs on gene expression in response to specific cues or external stimuli such as tobacco product use [37,38].

### LncRNAs Classification, Modes of Action, and Regulation in Health and Disease

Although consensus on how to classify lncRNAs is yet to be reached [102], one widely employed method of classification is based on the genomic position of lncRNAs relative to other genes, e.g., protein-coding genes [91]. Accordingly, lncRNAs are classified into three categories. (I) Intronic lncRNAs are transcribed from the intron of a sense or antisense gene; (II) intergenic lncRNA genes do not overlap with other genes; and (III) antisense or divergent lncRNA genes either overlap or are in close proximity to a sense gene and are localized on the opposite DNA strand [91,92,102]. Another method of classification relies on the mode of action and regulation of lncRNAs; these include but are not limited to target gene regulation through either *cis* or *trans* action [91]; molecular role, e.g., enhancer RNAs [109], competitive endogenous RNAs [110], and architectural RNAs [111]; transcriptional regulation, e.g., damage-induced lncRNAs [112] and stress-induced promoter-associated antisense lncRNAs [113]; or physiological relevance, e.g., angio-LncRs (*MALAT1*, *MANTIS*, *PUNISHER*, *MEG3*, *MIAT*, *SENCR*, and *GATA6-AS*) [114]. The process of transcription per se has also been suggested to give an additional dimension to the original function of lncRNAs [28]. For example, some lncRNAs may not be exclusively non-coding, and while retaining their original function, they may also give rise to small functional peptides otherwise known as micropeptides [115]. Alternatively, some lncRNA loci may be part of a 3D nuclear construct permissive to the chromatin environment and gene regulation at the neighboring loci [116]. LncRNAs can be found anywhere in the cell, although the majority of lncRNAs are localized to the nucleus, which may stem from inefficient splicing events [27]. Unlike mRNAs, most lncRNAs have low expression levels, are evolutionarily less well conserved, and exhibit high cell-type specificity or tissue specificity [28]. Promoter regions of lncRNAs contain fewer TF binding motifs [115]. LncRNAs can form secondary and tertiary structures and contain functional RNA elements and nuclear localization sequences, which are key components of gene regulation [27,117]. Together, these features enable lncRNAs to serve a wide range of regulatory functions in physiologic and pathologic conditions [115]. For example, in mammals, lncRNAs have been demonstrated to be instrumental in processes such as the p53-mediated response to DNA damage [118], V(D)J recombination and class-switch recombination in immune cells [119], cytokine expression [120], endotoxic shock [121], inflammation and neuropathic pain [122,123,124], cholesterol biosynthesis and homeostasis [125,126], growth hormone and prolactin production [127], glucose metabolism [128,129], cellular signal transduction and transport pathways [130,131,132], synapse function [133,134], and learning [135]. A growing body of research shows the importance of lncRNAs in the regulation of genes involved in disease pathogenesis [102]. Altered expression of lncRNAs has been demonstrated in a wide variety of diseases ranging from cancer to cardiovascular, respiratory, and immune diseases, among others [29,30,31,32,33,34,35,36].

## 8. LncRNAs in Vaping Research

Aberrant expression of lncRNAs has been demonstrated in biospecimens from e-cig users [13,44] and cells treated in vitro with e-cig aerosol extract [45]. Le et al. [45] performed microarray expression analysis on human induced pluripotent stem cell-derived endothelial cells (iPSC-ECs) treated in vitro with menthol-flavored e-cig aerosol extract. The iPSC-ECs were generated from four healthy donors. There were 183 upregulated and 297 downregulated lncRNAs together with 132 upregulated and 413 downregulated mRNAs in the treated iPSC-ECs. Co-expression network analysis of the top five upregulated and downregulated lncRNAs and the associated 412 differentially expressed mRNAs revealed that the downregulated lncRNAs were associated with genes participating in fatty acid metabolism, cell cycle, cell division, and cell adhesion, while the upregulated lncRNAs were associated with genes involved in iron ion binding, protein binding, and proton-transporting ATPase activity. Small interfering RNA (siRNA) knock down of lncRNA *LUCAT1*, which was significantly upregulated in the treated cells, led to attenuation of the enhanced cell permeability and reactive oxygen species (ROS) production while partially restoring cell migration ability [45].

Kaur et al. [44] compared the expression profiles of lncRNA in plasma-derived exosomes obtained from e-cig users, cigarette smokers, waterpipe smokers, dual smokers (both cigarettes and waterpipe), and non-users. Six to eight subjects were studied per group. The GeneChip^TM^ WT Pico kit was used for expression profiling. Differentially expressed lncRNAs were detectable in various groups as compared to non-users as follows: e-cig users (13 lncRNAs: 8 upregulated and 5 downregulated), cigarette smokers (7 lncRNAs: 2 upregulated and 5 downregulated), waterpipe smokers (18 lncRNAs: 9 upregulated and 9 downregulated), and dual smokers (9 lncRNAs: 4 upregulated and 5 downregulated). The differentially expressed lncRNAs in e-cig users vs. non-users were unique and did not overlap with those identified in other groups when compared to non-users. There were few overlapping lncRNAs that were commonly dysregulated across other comparison groups. Functional annotation of the differentially expressed lncRNAs by FunRich gene enrichment analysis showed significant enrichment for genes involved in steroid metabolism and steroid binding in e-cig users vs. non-users. The differentially expressed lncRNAs in other comparison groups were significantly enriched for genes involved in important biological processes, including cell differentiation and proliferation [44].

We performed RNA-seq analysis on total RNA isolated from oral epithelial cells of healthy adult “exclusive” vapers, “exclusive” cigarette smokers, and non-users (*N* = 42, 24, and 27, respectively) [13]. Our choice of oral epithelial cells for gene expression analysis was based on the following: (i) oral epithelium is the first site of “direct” exposure to known toxicants and carcinogens present in e-cig vapor and cigarette smoke [136,137,138,139,140]; (ii) oral epithelial cells are a major target for tumor development and other anomalies associated with tobacco use [141,142]; (iii) over 90% of all human cancers are of epithelial origin [143,144]; (iv) oral epithelial cells and lung epithelial cells show striking similarities in response to respiratory toxicants and carcinogens, as evidenced by the comparable patterns of genotoxic [145,146,147,148,149,150,151,152,153,154,155,156,157,158], epigenetic [159,160,161,162], and transcriptomic effects [163,164,165,166,167,168,169] in smokers’ oral cells and lung cells, respectively; and (v) oral epithelial cells are readily available for sampling by non-invasive techniques [136,166,170].

**Figure 1:**
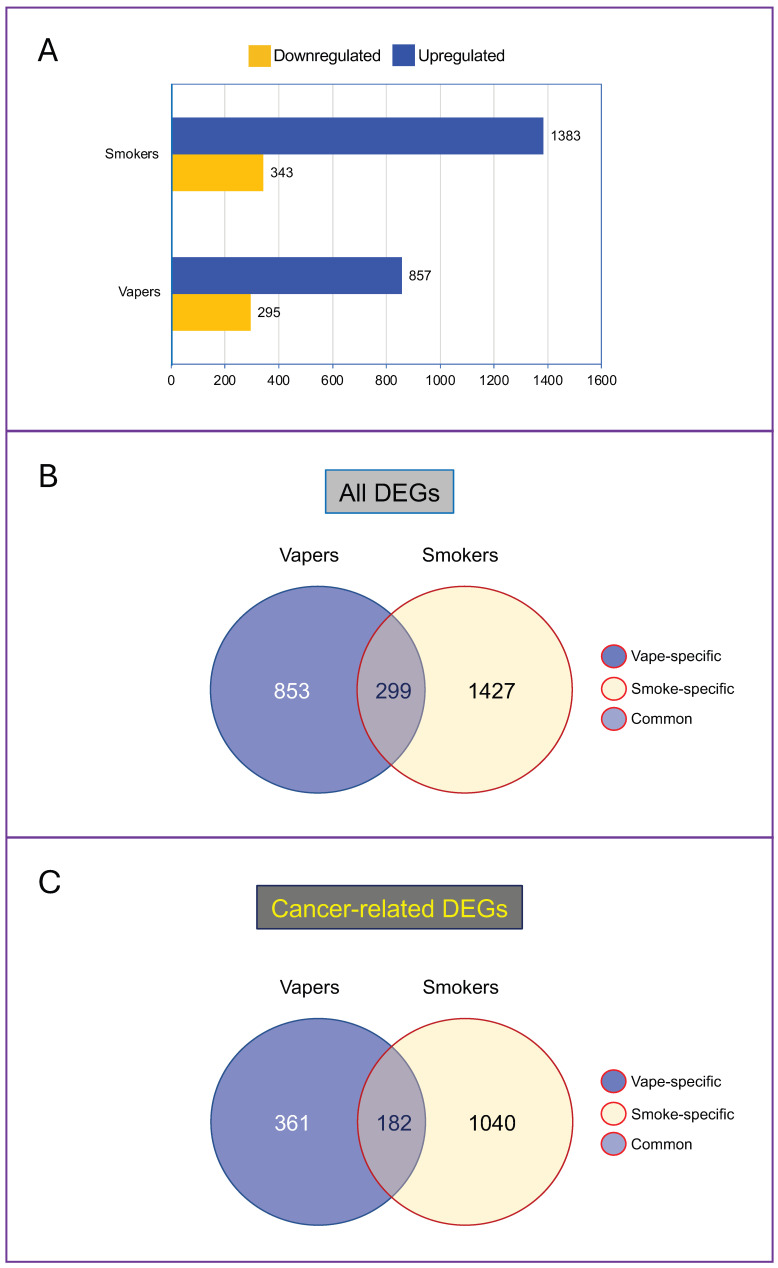
Aberrantly expressed genes in the oral epithelial cells of vapers and smokers as compared to non-users. (**A**) Numbers of upregulated and downregulated gene transcripts in vapers and smokers are indicated. Venn diagrams of all dysregulated genes (**B**) and cancer-related dysregulated genes (**C**) in vapers and smokers are shown. DEGs = differentially expressed genes. Data are from our previous publication (Ref. [13]). Detailed descriptions of data pre-processing, alignment, quantification, and differential expression analysis are provided in Ref. [13].

As shown in Figure 1A, there were large numbers of aberrantly expressed gene transcripts in both vapers and smokers as compared to non-users; however, smokers had nearly 50% more dysregulated genes than vapers (1726 vs. 1152). The aberrantly expressed genes in vapers and smokers can be divided into three categories: (I) vape-specific (853 transcripts); (II) smoke-specific (1427 transcripts); and (III) common to vape and smoke (299 transcripts) (Figure 1B). Gene ontology analysis revealed that cancer was the top disease associated with the dysregulated genes in both vapers (62%) and smokers (79%). The cancer-related dysregulated genes consisted of 361 genes specific to vapers, 1040 genes specific to smokers, and 182 genes common to vapers and smokers (total: 1583) (Figure 1C). The dysregulated genes in both vapers and smokers were also associated with other diseases and conditions, including inflammation. Of significance, some of the dysregulated genes in vapers and smokers are known to be frequently targeted in the early stages of diseases such as oral epithelial dysplasia, which can progress to malignancy [43].

**Figure 2:**
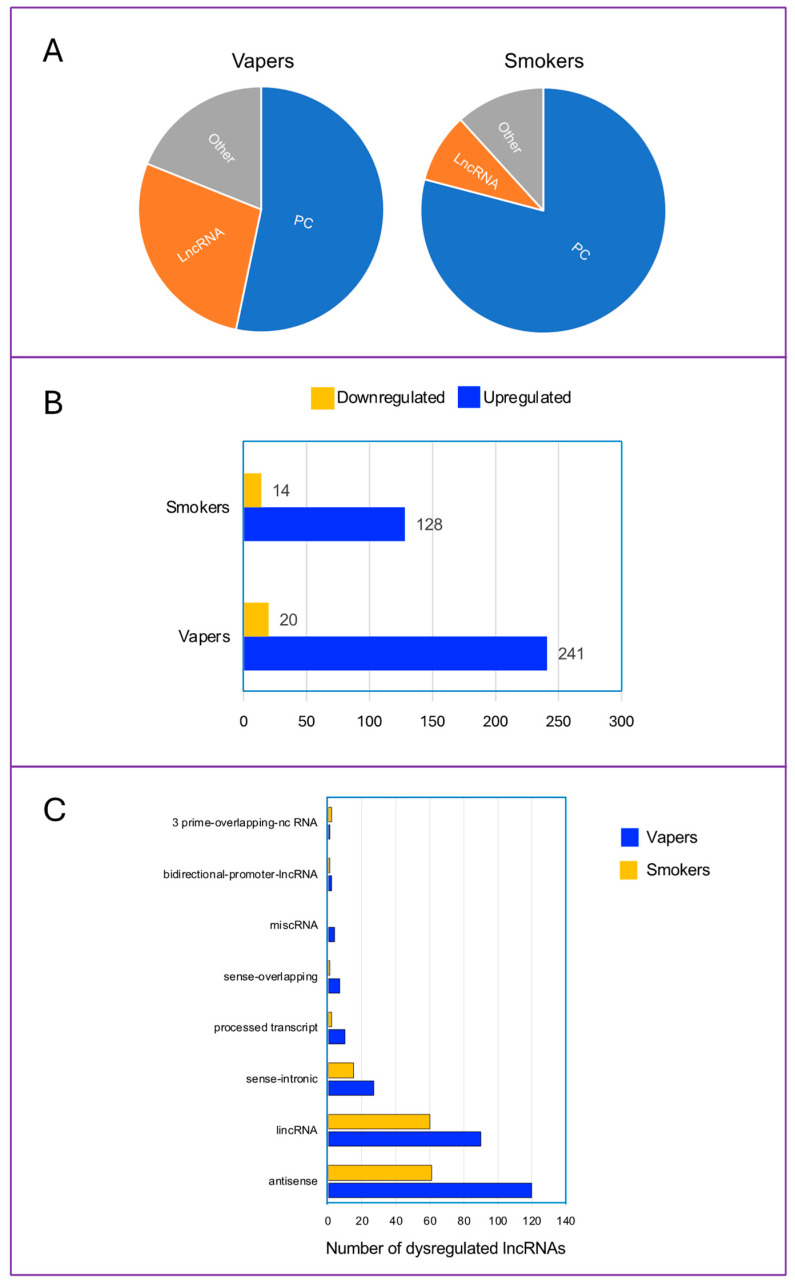
Classification of aberrantly expressed transcripts in the oral epithelial cells of vapers and smokers as compared to non-users. (**A**) Percentages of protein coding genes (PC), lncRNAs, and others (including (un)processed pseudogene, transcribed (un)processed pseudogene, to be experimentally confirmed (TEC), Ig V gene, rRNA, scaRNA, snoRNA, scaRNA/snoRNA, snRNA, and unitary pseudogene) are shown. (**B**) Numbers of upregulated and downregulated lncRNAs in vapers and smokers are indicated. (**C**) Sub-types of dysregulated lncRNAs in vapers and smokers. Total number of each sub-type of dysregulated lncRNAs is indicated. Data are from our previous publication (Ref. [13]). Detailed descriptions of data pre-processing, alignment, quantification, and differential expression analysis are provided in Ref. [13].

Importantly, 47% of the aberrantly expressed genes in vapers were non-coding (vs. 21% in smokers). Of these, 23% were lncRNAs in vapers (vs. 8% in smokers) (Figure 2A). More specifically, there were 261 and 142 differentially expressed lncRNA genes in vapers and smokers, respectively (*p* < 0.0001) (Figure 2B). The differentially expressed lncRNAs in vapers consisted of 241 upregulated and 20 downregulated lncRNAs, whereas in smokers, the respective numbers were 128 and 14 (Figure 2B). Figure 2C shows sub-types of the differentially expressed lncRNAs detected in vapers and smokers. There was no statistically significant difference in number of differentially expressed lncRNA of any sub-types between vapers and smokers.

Molecular pathway and functional network analysis of the dysregulated genes identified the “Wnt/Ca^+^ pathway” as the most affected pathway in vapers, while the “integrin signaling pathway” was most impacted in smokers. The Wnt/Ca^2+^ signaling pathway, which is activated by the tumor suppressor WNT5A in the presence of a “frizzled” class receptor, is downregulated in several types of human cancer [171]. Of significance, the WNT5A gene and the frizzled receptor FDZ7 gene were both downregulated in vapers, likely inhibiting the downstream effectors of the cascade. The integrin signaling pathway is shown to control cell proliferation, survival, and migration. When dysregulated, the integrin signaling pathway can promote tumor invasion and metastasis [172]. The “Rho family GTPases signaling pathway” was the most common dysregulated pathway in vapers and smokers, although the number of affected targets was three times higher in smokers than vapers (27 vs. 9). The GTPase family of small GTP-binding proteins comprises a group of signaling molecules that are activated by growth factors, hormones, integrins, cytokines, and adhesion molecules [173]. They regulate reorganization of the actin cytoskeleton, transcriptional regulation, vesicle trafficking, morphogenesis, neutrophil activation, phagocytosis, mitogenesis, apoptosis, and tumorigenesis. Rho GTPases are also implicated in the DNA-damage response following genotoxin treatment [173]. More in-depth examination of the dysregulated genes and integrative analysis of the lncRNA and mRNA data are currently underway in our laboratory.

## 9. Concluding Remarks and Future Directions

A growing body of research shows the importance of lncRNAs in regulation of genes involved in the development of human diseases [29,30,31,32,33,34,35,36]. LncRNAs are emerging as promising biomarkers for assessing exposure to disease-causing agents [40,42,174] as well as elucidating the underlying mechanisms of disease development [33,37,38,175]. A limited but burgeoning number of studies has shown the potential of lncRNAs for assessing the biological consequences of vaping [13,44,45]. The existing data show that lncRNAs mediate dysregulation of key disease-related genes in cells and tissues of vapers [13,44] and in cell treated in vitro with e-cig aerosol extract [45]. The dysregulated lncRNAs and associated molecular pathways and gene networks in e-cig users have been shown to be partly similar to and partly distinct from those found in smokers [13,44]. The aberrantly expressed lncRNAs common to vapers and smokers can be attributed to shared exposure of both groups to chemicals present in e-cig vapers and tobacco smoke [13,55,176]. On the other hand, the unique dysregulated lncRNAs specific to vapers or smokers are likely due to distinct exposure of each group to chemicals present only in e-cig vapor or tobacco smoke [13,46,176]. Dual users of e-cigs and combustible tobacco products exhibited dysregulation of some of the same lncRNAs that are aberrantly expressed in exclusive vapers and smokers [45]. Nonetheless, dual users have also shown unique dysregulated lncRNAs, which may be ascribed to the interactive effects of combined use of e-cigs and combustible tobacco products [45,67].

Thus far, the (relatively) small size of the studied populations has precluded examination of the contribution of product characteristics, e.g., e-cig device type or features and e-liquid content (i.e., flavor type, nicotine concentration, and humectants), to the observed effects in vapers [13,44]. Follow-up studies with large sample size and improved statistical power should determine the role of product characteristics in the lncRNA-mediated dysregulation of disease-related genes observed in vapers. Future investigations should consider the dynamic changes as well as uniformities in epigenetic marks across different cell types [11,12,159,177,178] when comparing the expression profiles of lncRNA in various biospecimens from e-cig users. In addition, the effect of local vs. systemic exposure to chemicals present in e-cig vapor should be taken into account when analyzing different cells and tissues from e-cig users, e.g., oral or nasal epithelia vs. peripheral blood.

Because many transcriptomic changes occur in the early stages of disease—often prior to clinical manifestation of the disease [179,180,181]—it is all but certain that one should detect dysregulated disease-related coding and non-coding genes, e.g., lncRNAs, in apparently healthy vapers, smokers, and dual users, as shown by us [13,23,176] and others [44]. The target lncRNAs/mRNAs in healthy vapers and/or conventional tobacco product users are likely to be dysregulated to a lower extent than those in patients diagnosed with diseases. Substantial and long-term exposure of chronic vapers and smokers to toxicants and carcinogens present in e-cig vapor and tobacco smoke should lead to transcriptomic changes including differentially expressed lncRNAs similar to those found in the patient population, although patients are likely to have more pronounced changes. The dysregulated lncRNAs in vapers and/or smokers have been associated with diseases like cancer, respiratory diseases, cardiovascular disease, and/or immune diseases [13,44,45]. This is consistent with fact that these diseases are most commonly caused by or linked to tobacco product use [1,3,5,8,10,15,23,65,176,182,183,184].

Since an individual lncRNA can have multiple mRNA targets [83,84,85,86], most dysregulated lncRNAs in vapers and/or smokers may be associated with multiple diseases. Of note, a biological pathway is rarely, if ever, affected in one disease only. To minimize noise and facilitate data interpretation, future investigations should use statistical and bioinformatic approaches to prioritize selection of lncRNA–disease pairs in vapers and/or smokers with the highest association specificity and sensitivity. Lastly, while association studies of molecular changes and disease are widely used for biomarker discovery in humans [179,185,186,187,188,189], follow-up functional studies involving RNA interference (RNAi) and antisense oligonucleotides [190,191,192,193] should be conducted to verify whether the dysregulated lncRNAs found in vapers and/or smokers can be causally linked to disease development.

## Data Availability

All data are contained within the article.

## Abbreviations

ceRNAs, competing endogenous RNAs; e-cig, electronic cigarette; ENDS, electronic nicotine delivery systems; FDA, United States Food and Drug Administration; iPSC-ECs, induced pluripotent stem cell-derived endothelial cells; lincRNAs, long intergenic noncoding RNAs; lncRNAs, long non-coding RNAs; miRNA, microRNAs; PG, propylene glycol; ROS, reactive oxygen species; siRNA, small interfering RNA; TF, transcription factor; VG, vegetable glycerin; VOC, volatile organic compounds
